# Health system capacity and infrastructure for adopting innovations to care for patients with venous thromboembolic disease

**Published:** 2014-04-01

**Authors:** Danielle A Southern, Jasmine Poole, Alka Patel, Nigel Waters, Louise Pilote, Russell D Hull, William A Ghali

**Affiliations:** Danielle A. Southern, MSc, is a Programmer/Analyst in the Department of Community Health Sciences and a member of the Institute for Public Health, University of Calgary, Calgary, Alberta.; Jasmine Poole is a Research Assistant in the Department of Medicine, McGill University Health Centre, Montreal, Quebec.; Alka Patel, PhD, is an Adjunct Assistant Professor in the Department of Community Health Sciences and a member of the Institute for Public Health, University of Calgary, Calgary, Alberta.; Nigel Waters, PhD, is a Professor in the Department of Geography and GeoInformation Science, George Mason University, Fairfax, Virginia.; Louise Pilote, MD, MPH, PhD, is a Professor in the Department of Medicine, McGill University Health Centre, Montreal, Quebec.; Russell D. Hull, MBBS, MSc, is a Professor in the Faculty of Medicine, University of Calgary, Calgary, Alberta.; William A. Ghali, MD, MPH, FRCPC, is a Professor in the Departments of Community Health Sciences and of Medicine and is Director of the Institute for Public Health, University of Calgary, Calgary, Alberta.

## Abstract

**Background:**

Diagnosis and treatment for venous thromboembolic disease (VTE) have evolved considerably through diagnostic and therapeutic innovations. Despite their considerable potential for enhancing care, however, the extent to which these innovations are being adopted in usual practice is unknown. We documented the infrastructure available in hospitals and health regions across Canada for provision of optimal diagnosis and therapy for VTE disease.

**Methods:**

Over the period January 2008 through October 2009, we studied health system infrastructure for care of VTE disease in Canada's 10 provinces and 3 territories and all 94 health regions therein. We interviewed health system managers and/or clinical leaders from all 658 acute care hospitals in Canada and documented key elements of health system infrastructure at the hospital level for these institutions.

**Results:**

There was considerable variation across Canada in the availability of key infrastructure for the diagnosis and management of VTE disease. Provinces with higher populations tended to have a large proportion of hospitals with capability to measure d-dimer levels, whereas less populated provinces were more likely to send samples to centralized analysis facilities for d-dimer testing. All provinces and territories had some facilities offering advanced diagnostic imaging, but the number of institutions and the availability of imaging were highly variable (with the proportion offering at least limited availability ranging from 0% to 90%). Only 6 provinces had regions with availability of dedicated early and/or long-term outpatient clinics for VTE disease.

**Conclusions:**

Infrastructure in Canada for optimal care of patients with VTE disease was suboptimal during the study period and was not entirely in step with the evidence. Such shortfalls in health system infrastructure limit the extent to which health care providers can deliver optimal, evidence-based care to their patients. Nationwide evaluations of health system infrastructure such as this one should be undertaken internationally to better characterize quality of care and potential for improvement.

Venous thromboembolic (VTE ) disease affects large numbers of Canadians each year through its 2 typical manifestations: deep vein thrombosis and pulmonary embolism. Given the high incidence of VTE disease (particularly among elderly patients),^1^–^3^ Canada's aging population, and the tendency for deep vein thrombosis and pulmonary embolism to behave as chronic or recurrent conditions,^4^ VTE disease has become one of the major drivers of overall hospital service use in Canada. The approach to diagnosis and treatment for VTE has evolved considerably, through diagnostic and therapeutic innovations. Specific innovations include the development of low-molecular-weight heparins^5^,^6^; medications that permit outpatient treatment for selected patients; new clinical prediction algorithms for estimating the probability of disease^7^,^8^; new blood tests for d-dimers^9^ (i.e., markers of clotting activity), which potentially enhance the diagnostic process; and new computed tomography (CT) scanning technology for detecting clots in both the lungs and the limbs, which potentially adds efficiency to the diagnostic process.^10^ For longer-term oversight of anticoagulation management, the creation of dedicated monitoring clinics has been associated with improved anticoagulation control. 11 All of these innovations have been endorsed by the American College of Chest Physicians and incorporated into its high-profile guidelines for diagnosis and management of VTE.^12^,^13^

Despite their considerable potential for enhancing care, the extent to which these innovations are being adopted in usual practice is not known. In particular, appropriate health system infrastructure is needed to achieve uptake by clinicians caring for individual patients and to permit optimal evidence-based care.

We undertook surveys and interviews of health system managers and targeted providers within regions and hospitals across Canada to document the extent of infrastructure for the following components of optimal care:

- diagnosis, specifically testing for d-dimers and various imaging modalities (computed tomography [CT], ventilation–perfusion scanning, venography, pulmonary angiography, and Doppler ultrasonography)
- therapy, specifically formulary access to lowmolecular- weight heparins, anticoagulation clinics, and critical pathways

Surveys and interviews were conducted both at the level of hospitals (specifically all acute care hospitals in Canada) and at the level of geographic health regions. The resulting work provides a transportable template for evaluating health system infrastructure in other countries and/or jurisdictions.

## Methods

We studied health system infrastructure for the care of VTE disease in 10 provinces and 3 territories in Canada and in each of the 94 defined health regions within these jurisdictions. We considered health regions as geographic units useful for the description and visualization of data on maps, not necessarily uniformly administered health systems for which care of VTE disease would be explained by a single administrative structure. We also targeted 658 individual acute care hospitals to document key elements of health system infrastructure at the hospital level. We surveyed acute care hospitals in Canada that provided acute care services to adults (i.e., individuals ≥ 18 years of age) and that had an emergency department.

### Study period and data collection

Data collection was performed by a nurse research assistant over the period January 2008 to October 2009. All acute care hospitals in Canada were contacted to identify stakeholders suitable to participate in our surveys and interviews. Initial contacts were identified through searches of region-specific websites or telephone directories. At initial contact with each health care organization, a brief description of the project was presented to the contact person, and a general inquiry was made to determine appropriate individuals to approach. We prompted the initial contact person, indicating that stakeholders of interest would be any of the following: managers of regional ambulatory care programs, managers of pharmacy and therapeutic services, administrative and clinical leaders of hospital emergency departments, or provincial contacts (for information about the provincial drug formulary). This prompting always generated at least one stakeholder contact and often yielded more than one. A multistep algorithmic procedure was followed to identify relevant stakeholders within each of the surveyed health regions and/or hospitals (details available from authors upon request). The individuals had to have knowledge of care and related services for patients with VTE disease. If identified stakeholders did not have such knowledge, we asked them to refer us to an alternate person. Stakeholder interviews, conducted by telephone in English or French, lasted from 10 to 30 minutes.

### Hospital-level data

We collected specific system structural characteristics at the level of individual hospitals representing the following key infrastructure elements that must be in place to permit clinicians to implement guideline-endorsed innovations for VTE disease care: availability of d-dimer assays in the hospital laboratories or as rapid bedside tests; availability of one or more specific types of d-dimer assays; existence of formal critical pathways to guide providers in the integrated use of clinical prediction algorithms, d-dimer assays, and diagnostic imaging; availability of specific diagnostic imaging modalities, such as ventilation– perfusion scanning, spiral CT scanning for pulmonary embolism protocols and CT venography, Doppler ultrasonography, venography, and pulmonary angiography; and hospital policies regarding availability of the above-mentioned imaging modalities after hours or on weekends.

### Region-level data

We simultaneously collected the following structural characteristics at the level of health regions: existence of outpatient follow-up clinics dedicated to oversight of early outpatient management of patients with VTE disease; existence of outpatient anticoagulation clinics and/or home self-monitoring programs for longer-term management of warfarin anticoagulation for patients with VTE disease; geographic locations of any existing outpatient follow-up clinics or anticoagulation management clinics; specific low-molecular-weight agents available regionally via the provincial or territorial formulary; and existence of accessible mechanisms to offset outpatient costs of expensive low-molecular-weight heparins for patients who do not have drug plans.

### Data analysis

We undertook simple descriptive analyses. For each element of health system infrastructure, we present overall Canada-wide data on the proportions of regions or hospitals with particular structural attributes. We present detailed tabulations on the proportions of regions and hospitals with each of the specific structural elements assessed.

We used geographic information systems to produce maps of Canadian health system infrastructure by region and by hospital. Each hospital location was geocoded by postal code using the Statistics Canada Postal Code Conversion File for 2006 within ArcGIS 9.2 software (Esri, Redlands, California). We then created maps showing the distribution of facilities with limited service (i.e., less than 24 hours a day and/or less than 7 days a week) and with 24/7 service for ventilation– perfusion scanning, CT scanning, Doppler ultrasonography, and pulmonary angiography.

## Results

Our overall findings indicated considerable variation across Canada in the availability of key elements of health system infrastructure required for optimal diagnosis and therapy of VTE disease. Our response rate was 100%, as we contacted every hospital and made frequent calls until a stakeholder could be interviewed.

### D-Dimer assays and hospital-based diagnostic imaging resources

**Figure 1:**
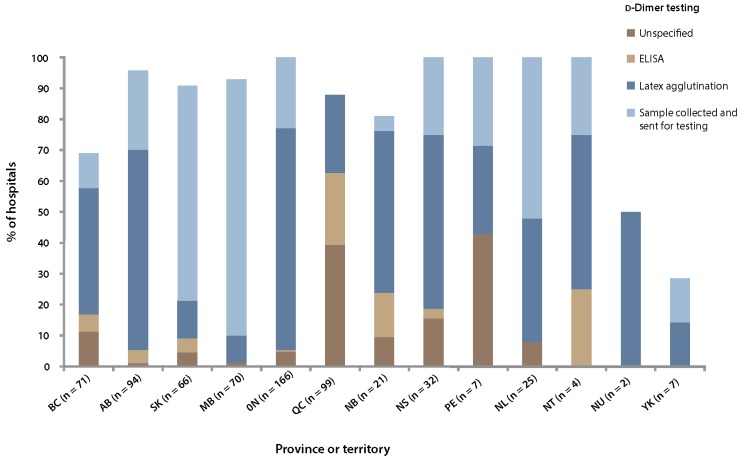
Availability and type of d-dimer testing available in 10 Canadian provinces and 3 territories. Unspecified, ELISA (enzyme-linked immunosorbent assay), and latex agglutination testing all refer to on-site testing.

The availability of testing for D-dimers or the collection and sending of samples for such testing varied widely across hospitals (Figure 1). In provinces with higher populations, most hospitals tended to have D-dimer testing available on site, whereas in provinces with smaller populations, hospitals were more likely to collect samples and send them to centralized facilities for analysis. In Yukon, there was minimal access to ddimer testing, either on site or through collection and sending to an off-site location for analysis.

There was also considerable variation across hospitals and jurisdictions in terms of hospital-based diagnostic imaging infrastructure (Table 1). All provinces had at least some facilities offering advanced diagnostic imaging, but the number of facilities and the extent of 24/7 availability was variable. The northern territories had limited availability of diagnostic imaging resources, either 24/7 or for limited hours. The proportion of hospitals with at least one of the VTE imaging modalities available (time limited or 24/7) varied across the provinces and territories, from a low of 0% (0/1 hospital) in Nunavut to a high of 90% (89/99 hospitals) in Quebec (Table 1).

**Table 1:** Availability of hospital-based diagnostic imaging resources in hospitals across Canada

Resource	Province or territory; no. (%) of hospitals												
	BC	AB	SK	MB	ON	QC	NB	NS	PE	NL	NT	NU	YK
No. of hospitals	71	94	66	70	166	99	21	32	7	25	4	1	2
V/Q scanning													
24/7	3 (4)	10 (11)	2 (3)	0	10 (6)	49 (49)	4 (19)	4 (12)	1 (14)	2 (8)	0	0	0
Limited	11 (15)	1 (1)	2 (3)	6 (9)	54 (33)	0	1 (5)	3 (9)	0	2 (8)	0	0	0
Spiral CT													
24/7	25 (35)	14 (15)	7 (11)	3 (4)	69 (42)	72 (73)	10 (48)	9 (28)	0	9 (36)	1 (25)	0	1 (50)
Limited	1 (1)	8 (9)	4 (6)	8 (11)	10 (6)	0	0	0	1 (14)	1 (4)	0	0	0
Doppler US													
24/7	23 (32)	13 (14)	11 (17)	4 (6)	52 (31)	87 (88)	8 (38)	12 (38)	1 (14)	11 (44)	0	0	0
Limited	16 (23)	17 (18)	2 (3)	13 (19)	95 (57)	0	4 (19)	5 (16)	1 (14)	2 (8)	3 (75)	1 (100)	1 (50)
Pulmonary angiography													
24/7	7 (10)	6 (6)	5 (8)	0	18 (11)	36 (36)	7 (33)	2 (6)	0	4 (16)	0	0	0
Limited	4 (6)	2 (2)	0	3 (4)	13 (8)	0	2 (10)	1 (3)	1 (14)	1 (4)	0	0	0
Venography													
24/7	7 (10)	12 (13)	10 (15)	1 (1)	15 (9)	30 (30)	5 (24)	6 (19)	0	9 (36)	0	0	0
Limited	10 (14)	11 (12)	0	10 (14)	45 (27)	0	6 (29)	3 (9)	1 (14)	1 (4)	0	1 (100)	1 (50)
Any imaging													
(24/7 or limited)	28 (39)	15 (16)	12 (18)	5 (7)	86 (52)	89 (90)	11 (52)	12 (38)	1 (14)	11 (44)	1 (25)	0	1 (50)

24/7 = 24 hours a day, 7 days a week; CT = computed tomography; "limited" = less than 24/7; US = ultrasonography; V/Q = ventilation–perfusion.

### Decision support tools

We found considerable variation in the number of hospitals with diagnostic critical pathways or other similar decision-support tools (Table 2). Most provinces had at least some hospitals where such tools were in use, but Prince Edward Island and the northern territories did not have facilities hosting such tools for VTE disease.

**Table 2:** Decision support tools available in hospitals across Canada

Province	Total no. of hospitals	Resource; no. (%) of hospitals	
		Diagnostic critical pathway	Computer prompts
British Columbia	71	12 (17)	1 (1)
Alberta	94	39 (41)	10 (11)
Saskatchewan	66	5 (8)	0
Manitoba	70	1 (1)	0
Ontario	166	40 (24)	7 (4)
Quebec	99	41 (41)	2 (2)
New Brunswick	21	16 (76)	2 (10)
Nova Scotia	32	15 (47)	0
Prince Edward Island	7	0	0
Newfoundland and Labrador	25	3 (12)	0
Northwest Territories	4	3 (75)	0
Nunavut	1	0	0
Yukon	2	0	0

### Diagnostic imaging

**Figure 2:**
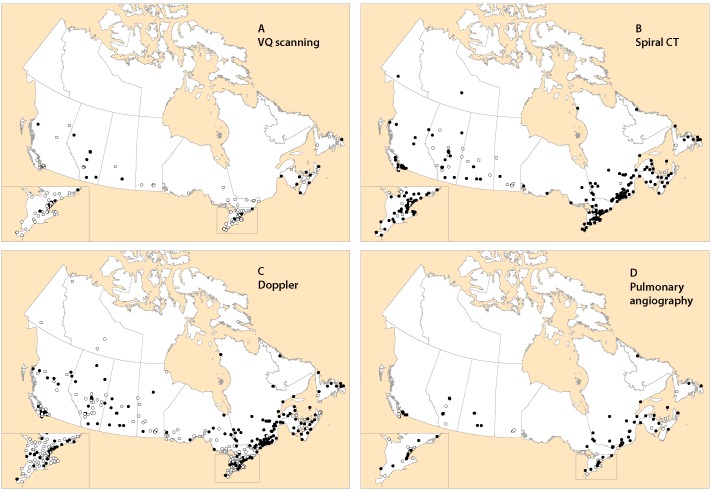
Availability of hospital-based diagnostic imaging services across Canada. Clear dots = limited services. Solid dots = service present 24/7. CT = computed tomography, VQ = ventilation–perfusion, 24/7 = 24 hours a day, 7 days a week.

Ventilation–perfusion scanning (Figure 2A) and pulmonary angiography (Figure 2D) were primarily available in the larger urban centres across the country. In contrast, CT scanning (Figure 2B) was more widely available across the country, with most locations offering 24/7 service. Although Doppler ultrasonography was available throughout the country, service was limited in many areas (Figure 2C).

### Outpatient clinics and home monitoring programs

Only 6 provinces had regions with dedicated early outpatient clinics and long-term outpatient clinics for VTE disease (Table 3). Dedicated home monitoring programs for VTE disease were even less available, with only 4 of the 94 regions reporting this type of service.

**Table 3:** Regional resources and clinics

Province	Total no. of regions	Resource; no. (%) of regions			
		Early outpatient clinics	Long-term outpatient clinics	Home programs	Home monitoring
British Columbia	5	0	0	0	0
Alberta	9*	5 (56)	4 (44)	2 (22)	0
Saskatchewan	13	2 (15)	1 (8)	0	1 (8)
Manitoba	11	1 (9)	1 (9)	0	0
Ontario	14	0	0	0	0
Quebec	18	17 (94)	17 (94)	0	0
New Brunswick	7	2 (29)	3 (43)	1 (14)	0
Nova Scotia	9	2 (22)	1 (11)	1 (11)	0
Prince Edward Island	1	0	0	0	0
Newfoundland and Labrador	4	0	0	0	0
Northwest Territories	1	0	0	0	0
Nunavut	1	0	0	0	0
Yukon	1	0	0	0	0

* At the time of data collection.

## Interpretation

This study was a significant undertaking in terms of data collection, with all 658 Canadian acute care hospitals with emergency departments contributing data to our survey. The findings reveal that infrastructure for VTE care innovations is present in Canada and that new diagnostic and therapeutic strategies are becoming available. However, their availability is not universal. Our survey was undertaken in 2008 and 2009, several years after new evidence emerged on the diagnostic and therapeutic innovations that we assessed, indicating a considerable lag in infrastructure becoming available after the appearance of supporting evidence. Our findings highlight the non-uniformity of Canada's health care system and the challenge of providing optimal care (and optimal infrastructure for care) to all residents of this geographically expansive country. Health system infrastructure is a necessary prerequisite to optimal processes of care, and it is only when both structure and process are optimized that optimal outcomes can be achieved. The approach that we took to generate these findings provides a template for the conduct of large-scale assessment of health system infrastructure.

Our finding of variation in the availability of health services across the country is not entirely new. Much previous research has discussed the concept of small area variation, with the conclusion that health information about a total population is a prerequisite for decision-making and planning in health care.^14^ Variations in care often arise from overutilization of some services in high-use areas, a scenario of supplysensitive care. Supply-sensitive care can be defined as any intervention for which the frequency of use depends largely on the availability of the resource to the patient.^15^ There has been much research into the relationship between geography and access to health care in the fields of cancer care and cardiac care.^16^ VTE disease care is somewhat different from both cancer care and cardiac care, because a larger pool of providers is involved. Also, VTE disease care is less often managed through referral to centralized tertiary care centres. Aside from pulmonary angiography, most of the imaging and testing required for VTE disease care are not tertiary services. Yet across the country, there is non-uniform availability of diagnostic and therapeutic infrastructure that may be limiting providers' ability to deliver optimal, evidence-based care. Ultimately, this is a health care policy issue that should be considered by health system decision-makers in the context of health care resource planning.

The availability of infrastructure for VTE disease care is an example of the Donabedian model. Donabedian, widely acknowledged as one of the leading thinkers in health services research, introduced a model for measuring quality based on system theory in 1966.^17^–^19^ According to this theory, health care is a system with objectives and components. In Donabedian's framework, the 3 components of health care quality are structure, process, and outcome. The structure is the environment in which health care is provided; it includes factors such as the ready availability of wellorganized primary care services, material and health resources, operational factors, and organizational characteristics of health care facilities (all of which are relatively stable). The process is the method by which health care is provided; it includes the giving of care by the provider and the receiving of care within the health care system. The outcome is the consequence of health care, including the health status of patients and communities. According to the Donabedian model, processes are constrained by the structures in which they operate, and it is this latter point that is underlined by the non-uniform availability of services revealed in this study. To the extent that some jurisdictions and hospitals have incomplete availability of services, the considerations and/or decisions around care of patients relate to either increasing locally available services, increasing hours of availability, or ensuring that patient transfer mechanisms are in place for regionalized care delivery.

### Limitations

Our study had some limitations. First, data collection for the entire country was a major undertaking. We acknowledge that some changes in availability of infrastructure may have been implemented since late 2009. It is even possible that our data collection process triggered some local actions (given that, anecdotally, a number of the individuals surveyed expressed interest in initiating quality improvement interventions toward the end of our study interviews). Second, we did not assess locally available transfer mechanisms. Patient transfer mechanisms can mitigate some of the challenges associated with lack of locally available infrastructure. Notably, however, we assessed availability of services in all acute care hospitals and health regions in this Canada-wide study. Given these important limitations, our results should be construed as representing a baseline assessment that identifies local opportunities for health system decision-makers to undertake infrastructure enhancement that could produce better processes, which in turn could lead to better outcomes. For front-line providers, this study provides information that can inform their advocacy for local system enhancements and associated quality improvement. A final caveat is that our study did not focus on outcomes, but rather on the Donabedian construct of health system structure. We have already explained that appropriate infrastructure is a prerequisite for optimized processes of care, which in turn can produce better outcomes. Future studies extending this work on VTE disease care could focus on process and outcomes.

### Conclusion

We comprehensively documented the extent of infrastructure available for optimal diagnostic and therapeutic care of patients with VTE disease across Canada. The full suite of infrastructure elements assessed in our study can serve as an infrastructure "checklist" of sorts for local health systems looking to implement innovations in VTE disease care that are in step with evolving practice guidelines. There are geographic inequalities in service availability for VTE across hospitals, which have downstream implications for process and outcome. Our findings underline how process of care and eventual optimized care are constrained by the system's infrastructure. Care innovations such as those for VTE disease challenge health systems to remain in step with evidence for optimal disease care.
